# Mast cell tissue heterogeneity and specificity of immune cell recruitment

**DOI:** 10.3389/fimmu.2022.932090

**Published:** 2022-07-29

**Authors:** Peter W. West, Silvia Bulfone-Paus

**Affiliations:** Lydia Becker Institute of Immunology and Inflammation, School of Biological Sciences, Faculty of Biology, Medicine and Health, University of Manchester, Manchester Academic Health Science Centre, Manchester, United Kingdom

**Keywords:** Mast cells, chemotaxis, cell heterogeneity, chemokines, cytokines, MRGPRX2

## Abstract

Mast cells occupy a unique niche within tissues as long lived perpetrators of IgE mediated hypersensitivity and anaphylaxis, as well as other immune responses. However, mast cells are not identical in different tissues and the impact of this tissue heterogeneity on the interaction with other immune cells and on defined immune responses is still unclear. In this review, we synthesize the characteristics of mast cell heterogeneity in the gut and the skin. Furthermore, we attempt to connect mast cell heterogeneity with functional diversity by exploring differences in mast cell-induced immune cell recruitment in these two model organs. The differential expression of certain receptors on mast cells of different tissues, notably tissue-specific expression patterns of integrins, complement receptors and MRGPRX2, could indicate that tissue environment-dependent factors skew mast cell-immune cell interactions, for example by regulating the expression of these receptors.

## Introduction

Beyond their classical role in IgE-mediated hypersensitivity reactions, mast cells (MCs) are now recognised to have diverse immunological functions (1). Their longevity as tissue-resident cells, particularly at barrier sites in the skin, intestine, lung and around blood vessels makes them uniquely situated to initiate, shape and resolve responses to insults, injury and infections (2). Additionally, we now appreciate MCs exhibit remarkable plasticity and specialisation such that, not only are their phenotype and responses shaped by their specific tissue environment in a steady-state, but also, the particular repertoire of preformed and synthesised mediators they release is context and stimulus-dependent (3). Here, we highlight current understanding of the differing nature of MC-induced immune cells recruitment, drawing on the examples of skin and intestinal MCs as archetypes of the traditional distinction between connective tissue and mucosal MCs.

## Mast cell mediator release

MCs of differing type and species produce a vast array of biologically active molecules including proteases, biogenic amines, cytokines, chemokines, growth factors and eicosanoids (4). While some of these products are stored in secretory granules, for near immediate release upon activation, others are newly synthesised and secreted within hours. Secretory granules consist of a dense gel matrix core formed of negatively charged proteoglycans such as heparin and chondroitin sulphate and amines such are histamine and serotonin as well as proteases, which typically, but variably include tryptases, chymases, carboxypeptidase A3, beta-hexosaminidase and cathepsins among others (5). Some cytokines including most notably TNFα, but also IL-4, IL-5, IL-13, IL-16, TGFβ, SCF, and growth factors, VEGF and NGF have been reported to be stored in MC granules in some circumstances (6–10). The release of granules, or their contents is tightly regulated and is an active process requiring calcium signalling, nucleoside catabolism and actin reorganisation (6).

Little is known about the cellular mechanisms of preformed mediator-storage in MCs apart from that of TNFα, which differs even between mice and human MCs, but involves transient exocytosis and re-endocytosis in humans (11). However, multiple different secretory vesicles and degranulation pathways exist suggesting a great deal of complexity in the control of secretion (12, 13). Apart from multigranular exocytosis, MCs also release smaller amounts of granule contents through piecemeal degranulation, kiss-and-run exocytosis, extracellular vesicle release and immune synapse formation. The release of *de novo* synthesised chemokines/cytokines likely differs from secretory granule mechanisms (6).

Whilst MC degranulation induced by FcϵR1α crosslinking, C3a, or substance P, results in immediate release, and later *de-novo* synthesis of chemokines/cytokines (14–16), activation of other receptors [reviewed elsewhere (1)] leads to more finely-tuned responses. For example IgE binding, in the absence of antigen-crosslinking, induces the release of IL-6 (17) and increases MCP-1 and CXCL8 release from IL-4-conditioned MCs (18). Weak, low receptor occupancy, FcϵR1α-crosslinking favours the production of chemokines, CCL2, CCL3 and CCL4 over cytokine production (19). Toll-Like Receptor (TLR) engagement results in the release of cytokines (*eg* IL-6) and chemokines in the absence of degranulation (1). MC responses are individualised to specific bacteria, where pathogenic *L.monocytogenes* induces degranulation and high levels of CXCL8 and MCP-1 release, while gut commensal *E.coli* induces lower levels of CXCL8 and MCP-1 without degranulation. Skin commensal *S.aureus* on the other hand does not induce CXCL8 or MCP-1 release and induces PGD_2_ at much lower levels (3). MC responses to viral infection include TNFα, type I interferon (20, 21), IL-4, IL-13, and chemokine production (22, 23). Purinergic receptor activation of MCs also releases cytokines (IL-6, TNFα, IL-33), leukotrienes, and chemokines (CCL2, CCL7, CXCL2) (24, 25).

The full plethora or MC mediators, cytokines, chemokines and their various roles are extensively reviewed elsewhere (1, 26). Those that induce immune cell recruitment and their cellular targets are listed in **Table 1**. MC-mediated activities that recruit leukocytes across tissues involves increasing vascular permeability (tryptase, histamine, VEGF, IL-6, CXCL8, LTC_4_, PGD_2_) (65, 66), upregulating adhesion molecule expression (TNFα) (26), recruitment of neutrophils (TNFα, IL-6, CXCL8, CXCL2) (51) monocyte/macrophages (TNFα, IL-6, CCL2) (67), ILCs (PGD_2_) (25), DCs (TNFα, GM-CSF, IL-1β) (68, 69), and CD8+ T-cells, (CCL5, LTB_4_) (4, 70). For example TNFα is stored and released by MCs from both skin and gut (71, 72), CCL2 is widely secreted and belongs to the core MC-transcriptional signature (44, 73, 74), and MCs have been shown to be essential for cell recruitment in many murine models (50, 51, 75). Mediator release is influenced by the inflammatory environment, for instance by IL-4, IL-5, IL-9, IL-33 and IFNγ (4, 76–79) which further regulate the expression of MC IL-2, IL-13, IL-9 and IL-25 (25, 29, 80–82). However there are tissue-specific aspects such as intestinal MCs lacking IFNγ (27) and skin MCs not producing LTC_4_ (57), which are yet to be fully elucidated.

**Table 1:** Key mediators and chemoattractants released by mast cells.

Mediator	Role	References
**Cytokines**		
**TNFα**	DC migration T-cell proliferation Monocyte Macrophage Activation Neutrophil recruitment	(27, 28)
**GM-CSF**	DC migration to lymph nodes	(29)
**IL-1**	Eosinophil recruitment Neutrophil recruitment DC-migration Inflammasome activation	(27, 29)
**IL-2**	T-regulatory cell recruitment ILC2 proliferation	(30, 31)
**IL-4**	Eosinophil recruitment Differentiation of T_h_0 cells to T_h_2	(32, 33)
**IL-3**	Mixed lymphocyte migration MC and basophil growth and differentiation	(29, 34)
**IL-5**	Eosinophil migration and survival	(35)
**IL-6**	Monocyte/Macrophage activation Local Neutrophil recruitment	(27, 29)
**IL-9**	MC proliferation DC migration T-cell recruitment	(36)
**IL-10**	CD8+ T-cell recruitment Inhibition of CD4+ T-cell recruitment Inhibition of proinflammatory cytokine production	(37–39)
**IL-12**	CD4+ Effector T-cell recruitment Th1 response induction Induction if IFNγ in NK, T_h_1, and MCs	(26, 40)
**IL-16**	T-cell recruitment DC migration	(27, 41)
**IL-17A**	B-cell recruitment (lung)	(42)
**IL-18**	DC migration	(27, 43)
**Chemokines**		
**CCL1**	Monocyte recruitment T-cell recruitment	(44)
**CCL2**	Neutrophil migration Monocyte migration T-cell recruitment MCp migration	(45)
**CCL3**	Monocyte recruitment T-cell recruitment	(44)
**CCL4**	Monocyte recruitment T-cell recruitment	(44)
**CCL5**	CD8+ T-cell recruitment Eosinophil recruitment Basophil recruitment Monocyte recruitment NK cell recruitment DC recruitment	(44)
**CCL7**	Monocyte recruitment T-cell recruitment NK cell recruitment Immature DC recruitment Basophil recruitment Eosinophil recruitment Hematopoietic progenitor cell recruitment	(46, 47)
**CCL18**	Naïve CD4^+^/CD8^+^ T-cell recruitment Memory T-cell recruitment B-cell recruitment Immature DC recruitment	(44, 48)
**CCL20**	B-cell recruitment Effector memory T-cell recruitment CD11b+ DC recruitment	(44, 49)
**CXCL1**	Neutrophil recruitment	(50)
**CXCL2**	Neutrophil recruitment Eosinophil recruitment Basophil recruitment	(44, 50, 51)
**CXCL3**	Neutrophil recruitment	(44)
**CXCL8**	CD4+ T-cell recruitment Neutrophil recruitment	(27)
**XCL1**	DC migration and cross presentation of antigen	(44, 52)
**CX3CL1**	Monocyte and T-cell recruitment and survival	(53, 54)
**Lipid Mediators**		
**LTB_4_**	Neutrophil chemotaxis DC chemotaxis MC chemotaxis CD8+ T-cell recruitment	(55, 56)
**LTC_4_**	Eosinophil migration	(57–59)
**LTD_4_**	Eosinophil migration Neutrophil migration	(60)
**LTE_4_**	Eosinophil migration ILC2 migration	(61, 62)
**PGD_2_**	Eosinophil chemotaxis and activation Basophil chemotaxis and activation T_h_2 cell recruitment & activation ILC2 migration Vasodilation Increased Vascular Permeability	(57, 63)
**PGE_2_**	DC migration	(64)

## Mast cell heterogeneity between and within tissues

The traditional categorisation of MCs based on histological findings into connective tissue (CTMC) and mucosal MCs (MMCs) in mice, and the human correlates of tryptase+ (MC_T_), tryptase+ and chymase+ (MC_TC_) and chymase+ (MC_C_) is well established (83–86). However, at transcriptional level, protease content exhibits greater tissue-specific heterogeneity both between and within tissues (73, 87). Additionally, while evolutionarily conserved, there are also differences between human and mouse mast cells (88).

One source of tissue heterogeneity is the MC embryonic origin. In both humans and mice, tissue MCs are seeded from MC progenitors originating from both embryonic yolk sac and adult bone marrow (89–91). During embryogenesis in mice, three waves of MCs are seeded successively from early erythromyeloid progenitors (EMPs), late EMPs and foetal hematopoietic stem cells. At birth, early EMPs-derived MCs constitute over 15% of MCs in pleural cavity, adipose tissue and skin, but less than 3% in gut and spleen. In contrast HSC-derived MCs were much higher in gut (>30%) than in skin (~8%) (89). In adult mice early EMP-derived MCs are maintained in the adipose tissue, a likely stem cell niche for MCs (92), but give way to other MC progenitors in skin (89, 90). Although sharing a core MC signature, these cells are transcriptionally distinct. Both embryonic and bone marrow-derived MCs, complete their maturation in peripheral tissues (89, 92). They are therefore subject to tissue-specific cues during that maturation process making them, to some degree, site specific. Additionally, in mice skin MCs are known to undergo *in situ* self-renewal by clonal expansion in the steady-state (93), whereas mucosal MCs are dependent on recruitment of MCp from the circulation (94–96). Nevertheless, both MMCs and CTMCs can be augmented by recruitment of MCp from the circulation during inflammation (93, 97, 98). Given the longevity of MCs this implies that environmental interactions during a lifespan can alter MC set points in the tissue which adds further complexity to MC heterogeneity.

A growing body of transcriptomic work demonstrates that MCs are distinct and highly variable between and within tissues. Indeed, MCs form a distinct transcriptional cluster differing greatly from other granulocytes. Furthermore, comparing between different types of connective tissue MCs, nearly 1000 genes can be differentially expressed (73).

Mouse MCs also display a marked tissue heterogeneity in their receptor profile (87). In both humans and mice TLR4 expression is low in skin, peritoneal and duodenal MCs but higher in colon and lung (99). Similarly, in mice the ATP receptor P_2_X_7_ is expressed more greatly in MCs from colon or lung rather than skin (24). C5aR1 expression also differs in some circumstances, in humans (100, 101). MRGPR receptor gene expression is a core component of murine foetal derived MCs (89) and tissue-resident MCs (73). However, intriguingly MRGPRX2, a receptor with wide agonist range and a key driver of pseudo-allergic reactions, shows large variation between tissues in humans (102). It is expressed at a high level in human skin, fat and synovial MCs, *i.e.* CTMCs. It is only expressed at low levels in lung and colonic MCs, which are refractory to substance P stimulation (57). Thus, this would suggest a distinct MRGPRX2-mediated response between mucosal and connective tissue.

Single cell RNA sequencing (scRNA-seq) across disease states in eosinophilic oesophagitis revealed a predominant tryptase and amphiregulin positive population of MC_TC_ in the lamina propria during homeostasis with the development of persistent epithelial localised chymase and cathepsin G high expressing MCs, and transient CD117high, ST2high population during active disease. In remission the transient locally proliferative population disappears, while the chymase population remains in the epithelium and the resident lamina propria MCs become CSF1high. These changes exemplify the plastic nature of human MCs which show both acute and chronic phenotypic alterations in response to tissue environment and spatial compartmentalisation. For example, MCs may switch from homeostatic to an inflammatory IL-33-responsive, and end with an IL-13-producing, macrophages supportive phenotype (103). Similar results have been obtained from scRNAseq studies in chronic rhinosinusitis in MC_T_ cells (CD117low, FcϵR1αlow), and MC_TC_ (CD117high, chymase^+^, cathepsin G^+^) from the epithelial and sub-epithelial compartments respectively. MC_TC_ expressed ST2, CSF1 and IL-13 in addition to CCL2, CCL3 and CCL4, whereas MC_T_ expressed TRAIL, FGL2 and IL-17RB. In this tissue, the authors discovered polarised states of a core set of MC genes that distinguished them from proliferative and unpolarised MCs and concluded that the polarised cells are primed to respond either according to a pro-inflammatory (MC_TC_) or a Th2-skewed pattern (MC_T_). Although similarities existed between MC_TC_ in this study and skin MC_TC_, the authors showed key differences in C5aR1 and MRGPRX2 expression, not only between the two populations but also within the lung (104). The continuous, rather than discrete, nature of human MC heterogeneity in the respiratory tract is supported by evidence from a large number of expressed cell surface proteins which show a continuous distribution in their expression (105).

Despite the marked transcriptomic heterogeneity observed by several authors, proteomic comparisons between two types of human connective tissue MCs, skin and fat, showed remarkably low levels of differentially expressed markers, at least in a quiescent state (102). There was also a remarkable interspecies correlation between mouse and human CTMCs and a common mast cell protein signature (102). Additionally, although mouse peritoneal MCs are reported to express the transcript for TLR4 (89), it was not found among 4620 MC expressed proteins during proteomic analysis (102).

In summary, discussions about the role of MCs in a given tissue drawn from general aspects of MC biology obtained across multiple tissues, cell lines or models are worthwhile and useful commentaries. However, one should challenge those views in light of the well-established tissue-specific heterogeneity of MCs and undertake a bigger effort in investigating MC-driven tissue-specific aspects of immunity.

## Mast cell-mediated immune cell recruitment in the gut

Situated throughout the gastro-intestinal tract, MCs are regarded as key in controlling organ homeostasis (106). Contributing to the maintenance of the epithelial barrier, the initiation and modulation of both innate and adaptive immune responses to pathogens, and the cross-talk with the enteric nervous system, MCs regulate tissue homeostasis and disease (107).

Intestine constitutive homing of MCp in mice, *via* transendothelial migration, is controlled by MC α4β7 integrin binding to adhesion molecules MAdCAM-1 and VCAM-1 (96) while chemoattraction is dependent on MC CXCR2 (108). Since germ-free mice have fewer intestinal MCs and express lower levels of CXCR2 ligands, this process is thought to be driven by commensal bacterial interactions with intestinal epithelial cells (109). In humans, intestinal MCs express mainly α2β1 integrin, which is not expressed in other mucosal or connective tissue MCs (110).

**Figure 1:**
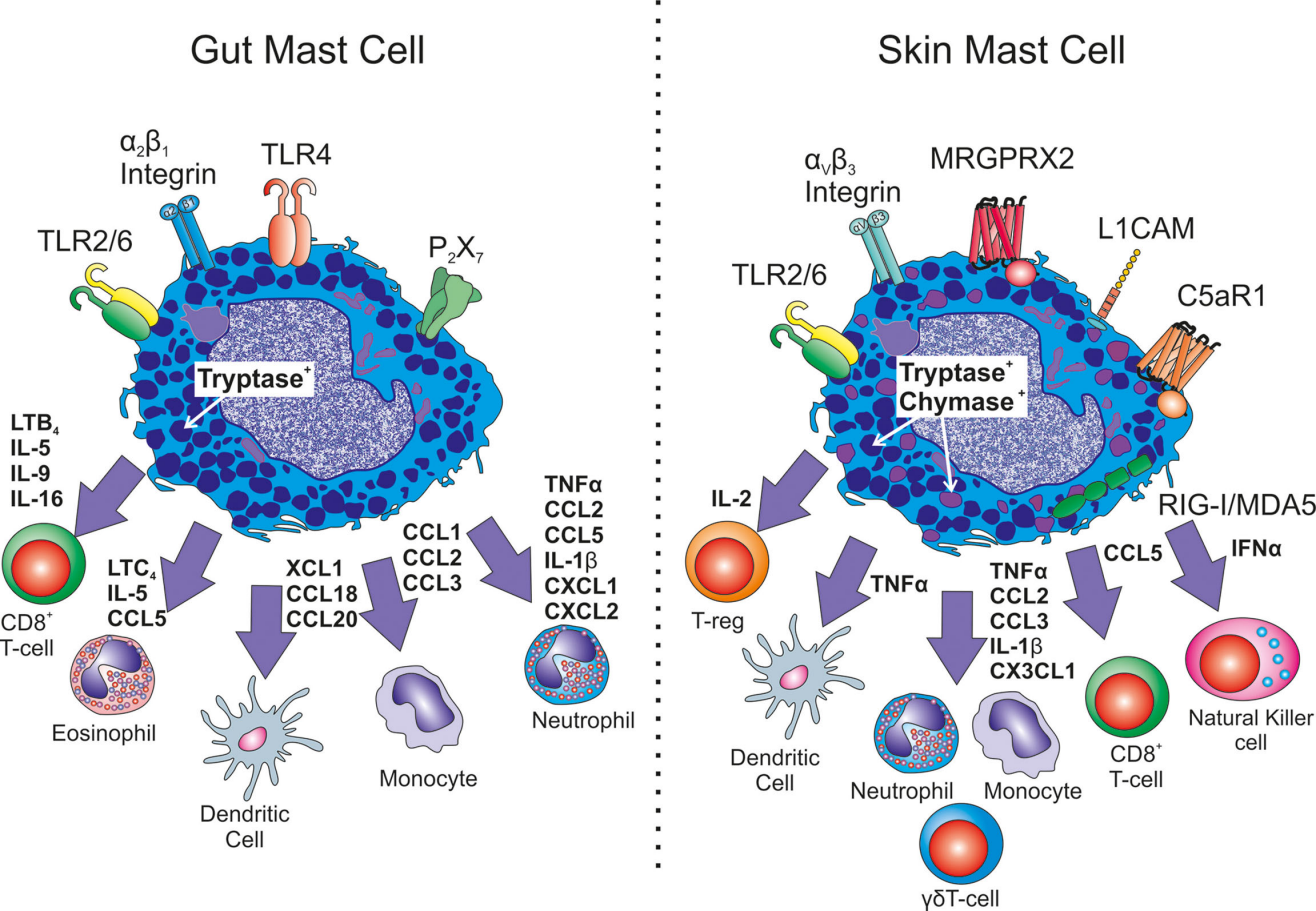
Heterogeneity in immune cell recrutiment. Exemplary differences between mucosal gut mast cells and connective tissue skin mast cells are shown. Differences in receptor expression include TLR4, P2X7, L1CAM, C5aR1, MRGPRX2 as well as integrin expression. Different mast cell mediators have been shown to be released with altered down stream consequences for immune cell recruitment in infection or inflammation.

MCs play a key role in the acute inflammatory response of the gut (see **Figure 1**). MC-derived TNFα recruits neutrophils, eosinophils and macrophages (94). Furthermore, human intestinal MCs produce an array of chemokines *de novo* when primed with SCF or IL-4 and activated by FcεRI-receptor crosslinking. These include CCL1, CCL2, CCL3, CCL4, CCL5, CCL18, CCL20, CXCL2, CXCL3, CXCL8 and XCL1 (44). Interestingly, CCL2, but not CXCL8, release by intestinal MCs in response to FcϵRIα activation exhibits a diurnal pattern indicating a role for MCs in intestine circadian biology (111). Moreover, in a gut injury mouse model, MC activation and Mcpt5, Mcpt6 and CPA3 protease release, is associated with neutrophil influx and alterations in epithelial barrier integrity. MC stabiliser cromolyn sodium results in preservation of barrier integrity, reduced neutrophil influx associated with significant reductions in TNFα, CCL2, CCL5, IL-1β and CXCL1 (112).

Direct evidence of MC involvement in anti-parasitic immunity of the gut is provided by a number of rodent models. *Trichinella spiralis* infection induces an influx of MC to the gut which transiently alters their protease expression. This is associated with a strong Th_2_ immune response, eosinophilia and marked increase in barrier permeability. The MC influx and the transiently-expressed MC-derived Mcpt1 is critical to control worm burden (113). Additionally, Mcpt6 is required for eosinophil recruitment (114). Mice lacking the proteoglycan serglycin, important in granule storage, also exhibit a deficit in worm clearance with lower circulating TNFα, IL-1β, IL-10, IL-13 compared to wild type infected controls (115). Human skin MCs also express these cytokines, and IL-13 downregulates T_h_1 responses in mice (116, 117), which raises the prospect that intestinal MCs could be a critical source of these cytokines in anti-parasitic immunity. Additionally Th_2_/Th_9_ cytokine IL-9, released early during parasitic infections and important for murine worm expulsion (118, 119) is also secreted by MC activated synergistically with LPS (120, 121), although the importance of MC-derived IL-9 in the intestine has not been proven definitively. In this regard, MCs in human gut are also immunoreactive for IL-5 and IL-16 both of which enhance T-cell recruitment and activation (26, 99, 122).

MCs have also been described to limit human colorectal cancers. In murine models MC-derived LTB_4_ was found to be critical for CD8+ T-cell recruitment and anti-tumor immunity (123).

## Mast cell-mediated immune cell recruitment in the skin

Like the gut, the skin is a barrier exposed to a wide array of environmental insults on a nearly continual basis. However, the skin is generally a less permeable barrier than the gut and as such MCs localise to the upper dermis around eccrine glands and blood vessels and not to the epidermis (124). Therefore, they regularly encounter fewer pathogens unless barrier integrity is disturbed through mechanical (*e.g.* wound or bite) or inflammatory means. This is thought to be a reason why TLR and P_2_X_7_ expression in skin MCs appears low, and maintains the cells in an unresponsive state (99). Low TLR4 expression might imply a diminished role for skin MCs in human skin Th9 biology (125). To our knowledge IL-9 has not been demonstrated in skin MCs. Nevertheless, skin MCs do respond to lipoteichoic acid, a TLR2 agonist component of commensal gram-positive bacterial cell walls, leading to enhanced responses to pox causing vaccinia virus in mice (126). Similar to recruitment in the gut, the skin microbiome is responsible for MC maturation. In this case through TLR2-dependent SCF production from keratinocytes (127) rather than direct interaction. In humans their adhesion is probably dependent on a different integrin αvβ3 (128). Large amounts of retinoic acid can also overcome the P_2_X_7_ down regulation in mouse skin MCs (129), emphasising the plasticity of tissue-resident cells.

Like other MCs, skin MCs have been identified to store and release TNFα in response to SP, FcϵRI, UVB and calcium ionohpore activation (130–132). There is good evidence from the murine passive cutaneous anaphylaxis model and delayed hypersensitivity reactions in skin, that leukocyte recruitment is dependent on MC-derived TNFα (51, 133), and that CD8^+^ dendritic cell migration to draining lymph nodes of the skin also relies on MC TNFα (68, 134) (**Figure 1**). Skin MCs can also exocytose TNFα containing granules which can be transported to the draining lymph nodes to enhance antigen presentation and antigen specific responses (135). This isn’t a phenomenon so far identified in other tissues.

MCs have recently been shown to have antiviral immune activities to dengue virus, a mosquito borne pathogen *via* activation of innate response pathway RIG-I/MDA5 resulting in TNFα, IFNα, CCL5, CXCL12 and CX3CL1 production. These MC-derived cytokines appear to be critical to limiting viral spread to lymph nodes through recruitment of NK and NKT-cells to the infected skin in a mouse model (53). Additionally, MCs form an immune synapse in infected skin with γδT-cells which induced activation through the T-cell receptor (136). There is also evidence that MCs are important in limiting other intradermal viral infections through DC activation (137). MC-derived TNFα and IL-6 have also been shown to be important for protection from cutaneous herpes simplex virus infection (138). In contrast, intestinal MCs are thought to have deficiencies in IFNγ production, and whilst RIG-I might be present in lung mucosal MCs it has not yet been identified in gut MCs (139).

The recently identified MC receptor for substance P MRGPRX2 and its murine homologue mrgprb2 (140, 141) has revealed a surprising dependence of a large degree of skin inflammation and pain on MCs. Green et al. (142) observed that Mrgprb2^-/-^ mice had significantly reduced hypersensitivity to inflammatory pain with not only ablated MC recruitment, but also reduced neutrophil and monocyte recruitment to the affected skin with a reduction in both CCL2 and CCL3 in response to injury or SP treatment of skin tissue. There has been much speculation about the involvement of gut MCs in painful symptoms of disease, however such compelling evidence is still lacking in other tissues and mucosal MCs have low MRGPRX2 expression.

In different murine models and human tissue samples, it has been demonstrated that skin MCs are sources of other cytokines with a likely impact on inflammation and immune activation. These include; IL-1β, which drives inflammasome activation and neutrophil recruitment in the skin (143, 144), an association also present in the gut (112); IL-2, not yet identified in gut MCs, which supresses inflammation in contact-hypersensivity through MC-dependent T-regulatory cell recruitment (30); IL-4 which drives type 2 inflammation in atopic dermatitis (32); IL-10 which as one of the first products of MCs, limit contact hypersensitivity reactions (37, 145) and also has a role in MC intestinal IBD pathology (146).

## Conclusions

Despite their heterogeneous origins, functionally, MCs from diverse tissues share a common set of responses including not least to IgE/allergen but also the release of key cytokines and chemokines involved in the initiation of inflammation *i.e*. TNFα, IL-1β, CCL2. MC plasticity then allows further functional differentiation based on the tissue context and lifetime environmental interactions such as pathogen or allergen exposure, or type I/type II inflammation. This plasticity will be shaped by existing heterogeneity in receptor expression such as C5aR1, MRGPRX2 and P_2_X_7_ which will limit the form of response that the cells can mount to a particular stimulus. For instance skin MCs might be better equipped to release IFNγ than intestinal MCs (27) whereas skin MCs will be more susceptible to neurogenic inflammation (57). Ultimately the application of new multi-omics, single cell and imaging mass cytometry technologies to isolated cells and tissues will reveal an as yet unforeseen level of detail about tissue-specific heterogeneity.

## Author Contributions

PW and SB-P reviewed literature and wrote the article. All authors contributed to the article and approved the submitted version.

## Funding

SBP is the recipient of a grant from MRC DPFS: MR/S036954/1. PW is supported by a GSK research grant. The funder was not involved in the collection of data, analysis, interpretation of data, the writing of this article or the decision to submit it for publication.

## Conflict of Interest

The authors declare that the review was conducted in the absence of any commercial or financial relationships that could be construed as a potential conflict of interest.

## Publisher’s Note

All claims expressed in this article are solely those of the authors and do not necessarily represent those of their affiliated organizations, or those of the publisher, the editors and the reviewers. Any product that may be evaluated in this article, or claim that may be made by its manufacturer, is not guaranteed or endorsed by the publisher.

## Abbreviations

FcϵRI, high-affinity IgE receptor; IL, interleukin; MCs, mast cells; MRGPRX2, Mas-related G-protein coupled receptor member X2; TNF, Tumor necrosis factor; TGF, transforming growth factor; SCF, Stem cell factor; VEGF, Vascular endothelial growth factor; NGF, Nerve growth factor; MCP, Monocyte chemoattractant protein; TLR, Toll-like receptor; PGD, Prostaglandin D; LTC, leukotriene C; GM-CSF, Granulocyte-macrophage colony stimulating factor; IFN interferon; CTMC, Connective tissue mast cell; MMC, Mucosal mast cell; EMP, Erythromyeloid progenitor; HSC, hematopoietic stem cell; CSF, Colony stimulating factor; TRAIL, Tumor necrosis factor-related apoptosis-inducing ligand; FGL, Fibrinogen-like protein; MadCAM, mucosal addressin cell adhesion molecule-1; VCAM, vascular cell adhesion molecule; RIG-I, retinoic acid-inducible gene I; MDA-5, melanoma differentiation-associated protein 5; NK, Natural killer; SP, Substance P.
